# Gene expression changes triggered by end-of-day far-red light treatment on early developmental stages of *Eustoma grandiflorum* (Raf.) Shinn.

**DOI:** 10.1038/srep17864

**Published:** 2015-12-08

**Authors:** Yoshihiro Takemura, Katsuou Kuroki, Masahiro Katou, Masayuki Kishimoto, Wataru Tsuji, Eiji Nishihara, Fumio Tamura

**Affiliations:** 1 Faculty of Agriculture, Tottori University, Koyama, Tottori 680-8553, Japan; 2 Tottori Prefectural Agriculture and forest Research Institute, Horticultural Experiment Center, Hokuei, Tottori 689-2221, Japan

## Abstract

To better understand the molecular mechanisms related to growth promotion in the early developmental stages of *Eustoma grandiflorum* (Raf.) Shinn. under end-of-day far-red light (EOD-FR) treatment, we analyzed the leaf transcriptome of treated (EOD) and untreated plants (Cont) by using RNA-seq technology. EOD-FR treatment for only about 2 weeks in regions with limited sunshine during winter resulted in significantly higher internode length between the 3rd and 4th nodes on the main stem in EOD than in Cont. Among the differentially expressed genes (DEGs) related to synthesis or transport of auxin, higher levels of *YUCCA* (CL6581) and *PIN4* (CL6181) were noted after treatment in EOD than in Cont in the leaf. In addition, high expression levels of *GA20ox* (Unigene11862) related to gibberellin (GA) synthesis and transcription factor *bHLH 135* (CL7761) were observed in the stem of EOD, 3 h after treatment. A vertical section of the stem showed that the pith length of cells at the 4th node was longer in EOD than in Cont. Collectively, these results suggested that EOD-FR treatment increased the expression of DEGs related to GA and auxin biosynthesis, *bHLH* transcription factor, and internodal cell elongation along the longitudinal axis of *Eustoma* plants.

*Eustoma grandiflorum* (Raf.) Shinn. is a quantitative long-day plant native to grasslands ranging from southern North America to northern South America^1^. *Eustoma* plants are very popular as an ornamental cut flower crop in Japan and are produced year-round in greenhouses in the warm western regions of Japan. However, in regions with limited sunshine during winter, retardation of plant growth and flowering is a serious problem for *Eustoma* production and subsequent shipments in winter and spring.

To promote stem elongation and flowering of *Eustoma* for winter and spring shipping, long-day treatments using incandescent lamps are employed^2^. A recent study found that fluorescent lamps emitting far-red (FR) light and incandescent lamps, each with a low R/FR ratio, promoted growth and flowering in *Eustoma* plants, whereas a daylight-type fluorescent lamp with a high R/FR ratio delayed growth and flowering^3^. Additionally, *Eustoma* plants grown under FR light for only 3 h at the end of day (EOD) during winter seasons showed early flower budding as well as longer main stem and higher node numbers than did untreated plants^4^. This method of promoting stem elongation by EOD-FR light treatment has been used for several plant species, including tobacco^5^, radish^6^, and soybean^7^. However, the effect of EOD-FR light treatment varied among cultivars of the same species; for example, EOD-FR light treatment of *Chrysanthemum* cultivars for 15 min, promoted stem elongation in Dekmona, Sei-elza, and Tourmalin, but it had no effect on stem elongation in Jimba^8^. Additionally, little is known about the molecular mechanism underlying plant growth promotion in ornamental cut flowers using EOD-FR light treatment.

Plant photoreceptors play an important role in FR light treatment. The most well known of these photoreceptors are phytochromes in leaf, which are soluble pigmented proteins that can exist in two spectrally distinct forms (phytochromes A [phyA] and B [phyB]) and sense ambient light conditions by photointerconversion between red and FR light-absorbing forms^9^. The contrasting roles of *phyA* and *phyB*, respectively, in sensing continuous FR and R lights indicate that these two members of the phytochrome family have discrete functions^10^. Mutant seedlings expressing *phyB* demonstrate attenuated responses to low R/FR ratio or to EOD-FR light, indicating that *phyB* plays a key role in shade-avoidance response^11^. phyB-deficient mutants of cucumber do not show increased stem elongation under natural radiation, indicating that *phyB* is required for the detection of the FR light component^12^. In addition, the *P*fr form of stable phytochromes influences their synthesis, catabolism, and sensitivity to gibberellins (GA)^13^. In cowpea, brief exposure to EOD-FR light results in increasing bioactive GA content and elongating epicotyls^14^. Moreover, GA is known to promote preferential cell growth along the longitudinal axis in rice (*Oryza sativa* L.), with a similar height phenotype conferred by loss-of-function mutations in a key GA biosynthetic gene, *OsGA20ox2*^15^.

On the other hand, the amount of auxin supplied also plays a role in stem elongation in pea (*Pisum sativum* L.)^16^. Among the various auxin response factors (ARFs) that bind to auxin and mediate auxin-induced gene activation, *ARF6* and *ARF8* are known to regulate growth in both vegetative and reproductive tissues, and arf6 and arf8 single mutants cause slight delays in stem elongation in *Arabidopsis*^17^. In wild-type *Arabidopsis thaliana*, Hornitschek *et al.*^18^ confirmed that low R/FR light treatment increased auxin concentration. Further, microarray analysis of *A. thaliana* leaf blades and petioles of plants subjected to EOD-FR light treatment revealed that almost half of the genes induced are auxin-responsive genes^19^. In addition, *PhyB P*fr photoconversion by FR light treatment inhibited NPH3 activity, which plays a role in establishing an auxin gradient along the adaxial–abaxial axis in *A. thaliana* leaves^20^,^21^. Moreover, these genes are regulated by the phytochrome in shade-avoidance response that is also related to the plant circadian clock. A recent study showed that EARLY FLOWERING 3 (*ELF3*) regulates developmental time and circadian clock period length in *Arabidopsis*, and the extent of this regulation is dependent on the light conditions^22^. At that time, Jiménez-Gómez *et al.*^22^ used a segregating population derived from two Arabidopsis ecotypes to investigate this variation and found a chromosomal region affecting the hade avoidance response. The growth-promoting effect of EOD-FR light treatment has been well studied in *E*. *grandiflorum* ‘Bolero White’^4^; therefore, elucidating the underlying molecular mechanisms by comparative analysis of treated and untreated individuals of this species will considerably aid the understanding of the effects of this treatment in many other ornamental cut flower species or cultivars. However, to the best of our knowledge, the genetic information of *Eustoma* spp. has not been studied thus far.

Microarray is a very useful method, but characterizing a large number of genes in a single experiment is difficult. On the other hand, RNA-seq technology is a highly efficient tool that allows fast gene discovery and accurate transcriptome profiling for gene characterization of species that lack reference genome information^23^. Here, we used RNA-seq technology to identify candidate genes related to growth promotion in the early developmental stages of *Eustoma* plants subjected to EOD-FR light treatment, and we compared the leaf transcriptome of treated and untreated plants. We specifically used the leaf tissue for the analysis, because the active phytochrome in R/FR light condition is a leaf transcription factor.

## Results

### Effects of EOD-FR light treatment on growth of E. grandiflorum

**Figure 1:**
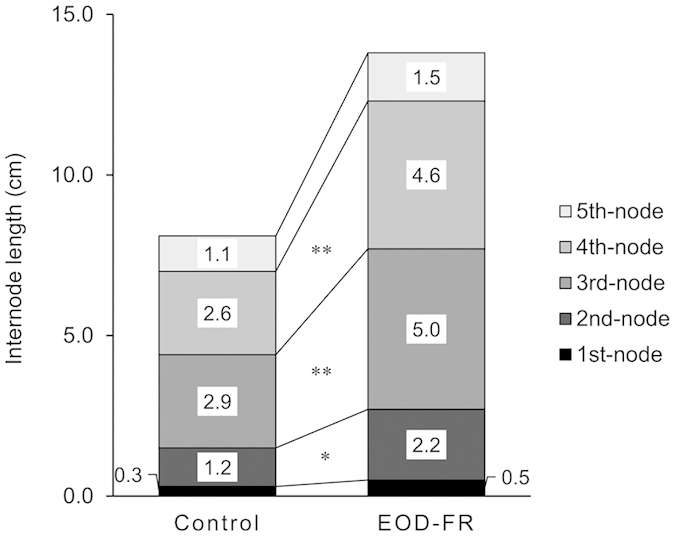
Internode length of the main stem in early developmental stages of *Eustoma grandiflorum*. Numbers in each column indicate the length of each internode. * and ** indicate significant difference with T-test at p < 0.05, or 0.01, respectively.

On December 25, stem and mean internode lengths in plants treated with EDO-FR light (EOD) were significantly greater than those of Control plants (Cont) (Table 1). However, no significant differences were noted in the number of nodes on the main stem and fresh weight of above and belowground biomasses in both treatments. Internode lengths of the 2nd, 3rd, and 4th nodes on the main stem of EOD were significantly longer than those of Cont (Fig. 1).

### Sequencing, de novo assembly, and identification of differentially expressed genes in EOD and Cont

RNA-seq generated 0.2 billion reads with approximately 6.5 billion nucleotides from each leaf sample collected from EOD and Cont, and a total of 161,051 contigs were assembled using clean reads (Table 2).

To identify the candidate genes involved in the growth promoting effect in *Eustoma* plants subjected to EOD-FR light treatment, we compared EOD and Cont by differential expression analysis. The transcript abundance of each gene, estimated as reads per kilobase of exons per million reads mapped (RPKM), showed 1,082 differentially expressed genes (DEGs), among which 602 DEGs were highly expressed in EOD and 462, in Cont (Supplementary Fig. S2; Supplementary Table S1).

### GO (Gene Ontology) analysis

Of the assembled unigenes, 103,985 (64.57%) were annotated using BLAST and successfully categorized into GO groups (Supplementary Fig. S1). In the biological process category, the largest groups were “cellular processes” and “metabolic processes.” In the cellular component category, the largest amount of genes was mapped to the terms “cell” and “cell part.” In the molecular function category, “binding” and “catalytic activity” were the largest groups.

GO enrichment analysis showed that most DEGs fell into the three broad GO categories of “biological process,” “molecular function,” and “cell component” with significance (Supplementary Fig. S3; Supplementary Table S2). Among the GO terms in DEGs upregulated by EOD-FR light treatment, the largest group was “oxidation–reduction process” in the biological process category (Table 3). “Transmembrane transport” and “regulation of transcription, DNA-dependent” were also large groups in the same category. In the molecular function category, “sequence-specific DNA binding transcription factor activity” was the largest group. In the cellular component category, the largest amounts of genes were mapped to the term “nucleus.”

### Kyoto Encyclopedia of Genes and Genomes (KEGG) analysis

The DEGs were also subjected to KEGG pathway enrichment analysis. Among all unigenes, 60,494 genes were annotated with the KEGG pathway analysis, whereas 577 genes among 1,082 DEGs were successfully annotated.

After KEGG enrichment analysis, 14 pathways were significantly enriched (P < 1E-05) (Supplementary Table S3). In addition, the KEGG pathway analysis showed enrichment of pathways involved in circadian rhythm, starch and sucrose metabolism, photosynthesis (Supplementary Fig. S4A, S4B and S4C; Supplementary Table S4), and plant hormone signal transduction (Supplementary Fig. S4D). The transcript abundance of genes involved in terpenoid backbone, flavonoid, flavone, and flavonol biosyntheses were higher in Cont than in EOD (Supplementary Fig. S4E, S4F, and S4G). In the diterpenoid biosynthesis pathways, higher transcript abundance was noted for genes involved in GA synthesis in EOD than in Cont (Supplementary Fig. S4H).

### Differential gene expression analysis by real-time PCR

**Figure 2:**
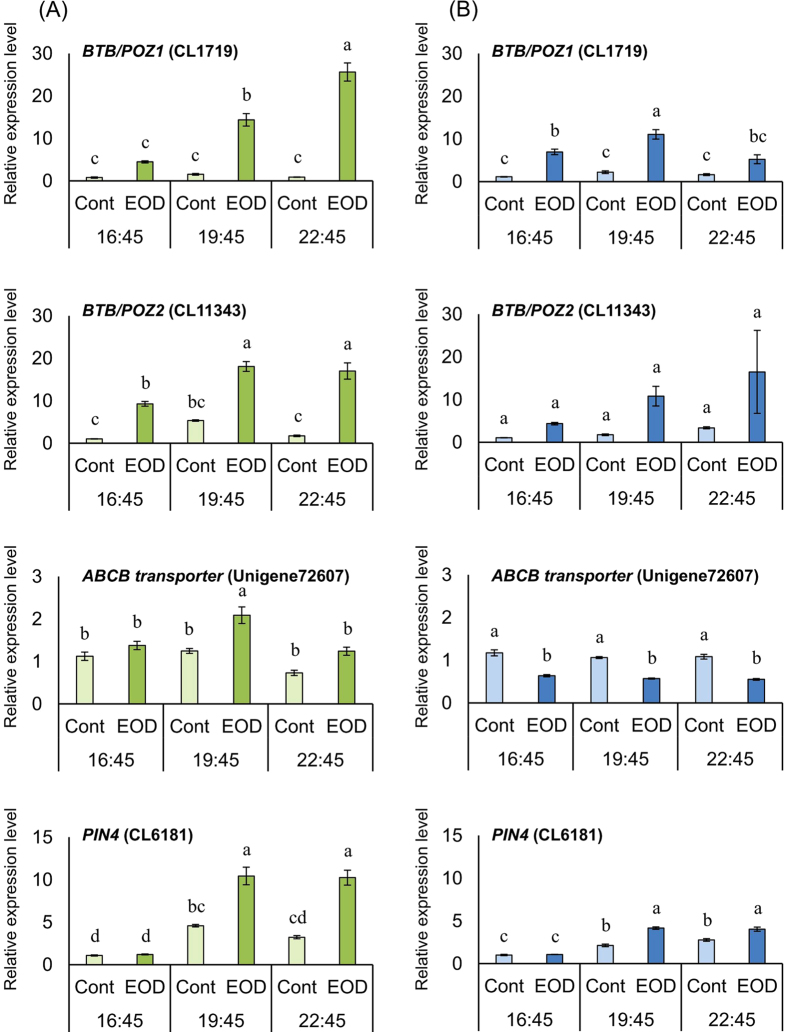
Relative expression levels of DEGs classified as “regulation of transcription, DNA-dependent” or “transmembrane transport” into the biological process category of GO terms in leaf (A) and stem (3rd- and 4th-nodes) (B) of *Eustoma grandiflorum* collected on December 25. Different letters within the same column show a significant difference by Tukey-Kramer’s HSD tests at the 5% level.

Relative expression levels in leaf and stem (3rd- and 4th-nodes) of *Eustoma* plant collected on December 25 were analyzed by real-time PCR. Among the DEGs classified as “regulation of transcription, DNA-dependent” in the biological process category of GO analysis, expression levels of *BTB/POZ1* (CL1719) and *BTB/POZ2* (CL11343) annotated as BTB/POZ domain-containing protein were higher in the stem and leaf of EOD than of Cont at 16:45, respectively (Fig. 2). Subsequently, the expression levels in the leaf and stem of *BTB/POZ1* and leaf of *BTB/POZ2* of EOD increased during 16:45–19:45; furthermore, the level of *BTB/POZ1* increased in the leaf but decreased in the stem at 22:45. Among the DEGs classified as “transmembrane transport” in the biological process category, the expression level of *ABCB transporter* (Unigene72607) was lower in the stem of EOD than of Cont at all sampling times. The expression level of *PIN4* (CL6181) was higher in EOD than in Cont in both the leaf and stem at 19:45 and 22:45.

Among the DEGs annotated as genes related to synthesis or signaling of auxin, the expression levels of *YUCCA* (CL6581) in the leaf was higher in EOD than in Cont at 19:45; furthermore, the level increased at 22:45 (Supplementary Fig. S5). This result was inconsistent with the observation in *AIP5* (Unigene42565), which had higher expression levels in stem of Cont than in EOD at all sampling times. *AIP15* (CL4296) expression was higher in EOD than in Cont at 22:45 in the leaf and at 19:45 in the stem.

**Figure 3:**
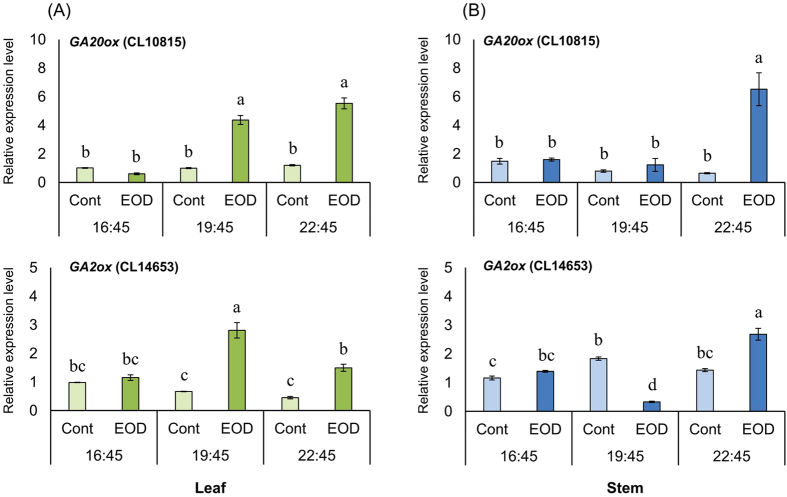
Relative expression levels of DEGs annotated as GA20ox and GA2ox in leaf (A) and stem (3rd- and 4th-nodes) (B) of *Eustoma grandiflorum* collected on December 25. Different letters within the same column show a significant difference by Tukey-Kramer’s HSD tests at the 5% level.

The DEGs *GA20ox* (Unigene11862) and *GA2ox* (Unigene15251) showed higher transcript levels in EOD than in Cont in the leaf at 19:45 and 22:45 (Fig. 3). Furthermore, the expression level of *GA2ox* in the leaf in EOD decreased during 19:45–22:45. In the stem, the expression level of *GA20ox* was higher in EOD than in Cont at 22:45, whereas that of *GA2ox* was higher at 19:45 but lower at 22:45 in Cont than in EOD.

**Figure 4:**
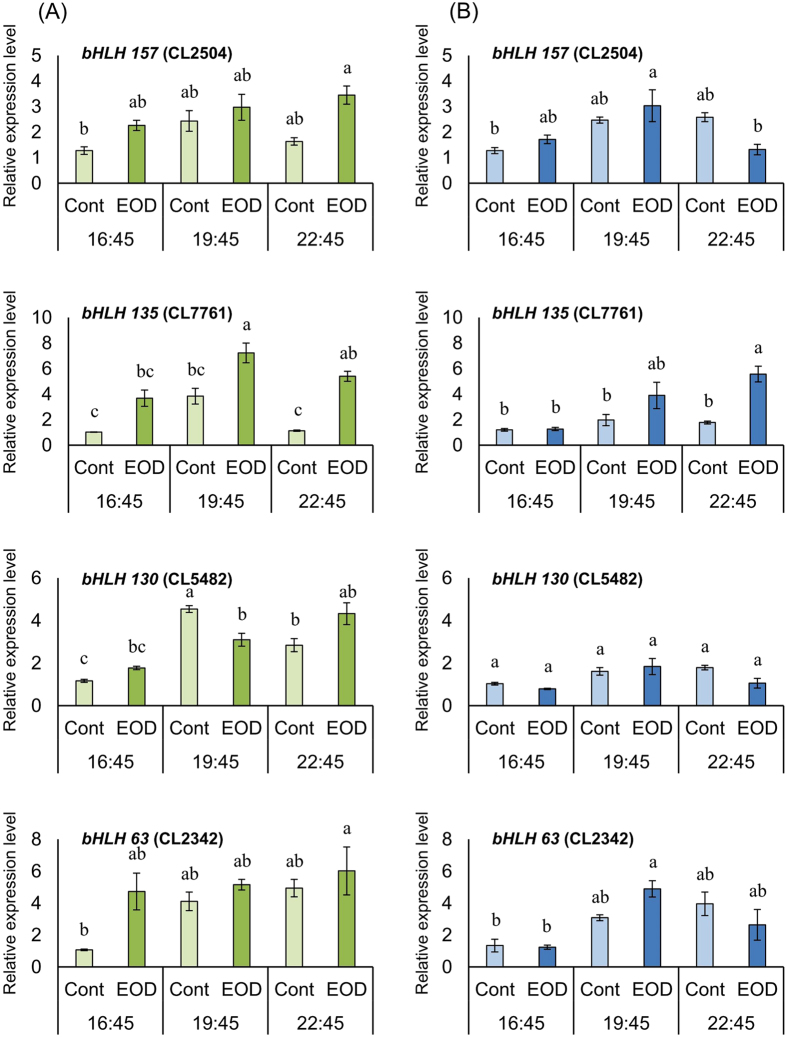
Relative expression levels of DEGs annotated as transcription factor bHLH up-regulated by EOD treatment in leaf (A) and stem (3rd- and 4th-nodes) (B) of *Eustoma grandiflorum* collected on December 25. Different letters within the same column show a significant difference by Tukey-Kramer’s HSD tests at the 5% level.

Among the DEGs annotated as transcription factor bHLH, compared to Cont, the expression levels of *bHLH 135* (CL7761) in EOD were higher in the leaf at 19:45 and 22:45, and in the stem at 22:45 (Fig. 4). In the leaf, the expression level of *bHLH 130* (CL5482) was higher in Cont than in EOD at 19:45, whereas the expression of *bHLH 63* (CL2342) was higher at 16:45 in EOD than in Cont.

### Morphology of the internode by using light microscopy

We compared the 3rd and 4th nodes that showed significantly different lengths (*t*-test, p < 0.01) between EOD and Cont and microscopically observed the cross and vertical section morphologies of the stem pith and cortex. In the cross section, cortex area and cell area at the 4th node had reduced in EOD (Supplementary Table S5). In the vertical section; however, the width of the cell was significantly greater in Cont than in EOD (Table 4). Similarly, at the 3rd node, significantly larger cortex area and cell area were noted in Cont and in EOD; however, the cell length into the pith of 4th node and into the cortex of 3rd-node was greater in EOD than in Cont.

## Discussion

In the field of horticultural science, EOD-FR light treatment has been applied to numerous plants as an artificial technique for promoting stem elongation or flowering of ornamental cut flowers, including *C. morifolium* R.^24^, *E. grandiflorum* R.^3^, *Antirrhinum majus* L.^8^, *Helianthus annuus* L.^8^, *Matthiola incana* L.^8^, and *Cucumis sativus* L.^25^. Our results show that stem length and mean internode length in *Eustoma* plants can be promoted by EOD-FR light treatment for about 2 weeks in regions with limited sunshine during winter. In addition, no differences noted in the number of nodes and significant differences in the internode length of mid-nodes (2nd, 3rd, and 4th nodes) on the main stem between treated and untreated plants suggest that EOD-FR light treatment promoted internode elongation rather than increasing the number of internodes during the early growth stages of *Eustoma*.

A previous study showed that EOD-FR light treatment promoted the activation of photosynthate allocation, thereby enhancing growth in *Eustoma* plants^4^; however, little is known about the candidate genes related to the growth promotion by this treatment. The phytochromes that sense R/FR light conditions play a key role in shade-avoidance response^11^. Additionally, phytochrome in the leaves is more important than that in the stem in determining the rate of stem elongation^26^,^27^. Therefore, we analyzed the leaf transcriptome of Cont and EOD at 19:45, which is end time of the EOD treatment. Here, we used RNA-seq technology to generate a total of 161,051 contigs from each sample. After assembling by clean reads (Table 2), 103,985 unigenes (64.57%) were annotated using BLAST and successfully categorized the GO groups (Supplementary Fig. S1).To our knowledge, genetic information of *Eustoma* plants is lacking and this is the first study to describe the use of RNA-seq technique to identify large numbers of genes in the leaf of *Eustoma*.

Further, 602 and 462 DEGs were found to be highly expressed in EOD and Cont, respectively (Supplementary Fig. S2; Supplementary Table S1). However, these DEGs were expected to not be directly involved in flower-bud formation, because the plants, after about one month from the start of treatment (January 22), did not yet show flower-bud formation (data not shown). Additionally, results of the GO analysis suggest that some DEGs are involved in EOD-FR light treatment-specific expression (Table 3). A comparative GO analysis of the biological process and molecular function showed that higher percentage of genes related to “regulation of transcription, DNA-dependent” and “sequence-specific DNA binding transcription factor activity” were upregulated during the EOD-FR light treatment. Almost all genes categorized under “regulation of transcription, DNA-dependent” were annotated as BTB/POZ domain-containing protein. Furthermore, EOD treatment increased the expression levels of *BTB/POZ1* (CL1719) and *BTB/POZ2* (CL11343) in the leaf. Previous studies have shown that *PhyB P*fr photointerconversion by FR light treatment promoted leaf curling in *A. thaliana* by inhibiting the leaf-flattening activity of NPH3, which include BTB/POZ-domain proteins and are known to interact with phytochrome transcription factors^20^,^21^. Accordingly, these studies proposed that BTB/POZ domain-containing protein expression was induced in EOD promoted conversion of *PhyB P*fr. Further, the induced high expression levels of BTB/POZ domain-containing protein in EOD were also maintained after treatment.

In addition, NPH3 regulates polar auxin transport as a downstream step in phototropic responses; polar-localized NPH3 proteins regulate polarity and endocytosis of *PIN*^28^,^29^. Auxin is exported from the cell by *PIN* and some members of the *ABCB* transporter family^30^,^31^,^32^. In this study, *ABCB transporter* (Unigene72607) and *PIN4* (CL6181) were also classified as “transmembrane transport” in the biological process category. The expression levels of *PIN4* in both leaf and stem were upregulated by EOD treatment. The expression of several members of the *PIN* family is upregulated by light, and in some growing plants, phytochromes contribute to this regulation and low R/FR modulates auxin distribution by altering the cellular location of *PIN*^20^,^33^. Stem elongation by auxin application depends on the amount of auxin supplied to the plant^16^. In addition, the mRNA levels of several *YUCCA* enzymes in the auxin biosynthesis pathway have been shown to increase under low R/FR^34^,^35^. Our study results also support the above findings that EOD-FR light treatment increased the expression levels of *YUCCA* (CL6581) and *AIP5* (Unigene42565) annotated as genes related to auxin signaling (Supplementary Fig. S4). Accordingly, we suggest that the activation, synthesis, and transport of auxin by EOD-FR light treatment affected growth promoting of *Eustoma*.

In addition, GA 20-oxidase biosynthetic genes involved in petiole elongation in *Arabidopsis* increased with exposure to EOD-FR light or long-day FR-rich incandescent lamps^36^. Similarly, results of KEGG pathway enrichment analysis in this study indicated that the transcript abundance of genes involved in the synthesis of GA in diterpenoid biosynthesis pathways were higher in EOD (Supplementary Fig. S4G). Our study results also indicate that the expression of DEGs annotated as *GA20ox* (CL10815) were upregulated in both leaf and stem by EOD treatment, whereas the expression of *GA2ox* (Unigene15251) was temporarily downregulated in the stem at 19:45. In *A. thaliana*, the transcript levels of *GA2ox* as a GA-deactivating gene decreased by Pr under R light treatment^37^,^38^. Further, after 24 h blue light treatment, it was indicated that the circadian rhythmic expression of *GA2ox* was markedly different in plants grown under long-day and short-day photoperiods^39^. Therefore, we confirm that the increased *GA2ox* expression in the leaf of EOD involved phytochrome deactivating in FR light treatment and *GA2ox* expression pattern difference in the stem in EOD and Cont, which induced changes in the circadian rhythm. Further, our results suggest that the high expression level of *GA20ox* (over 10-fold more than that of Cont) had an effect on the increase of GA content in the stem. Previous studies have also shown increase of bioactive GA content and elongating epicotyls of cowpea by brief EOD-FR light treatment, petiole and stem elongation and increase in GA content in *Arabidopsis*, bean and spinach^14^,^40^,^41^. In addition, inhibition and promotion of stem elongation has been reported in *Eustoma* plants treated with uniconazole-P and prohexadione-calcium as a GA synthesis inhibitor, and GA1, respectively^42^. Hormonal signals, including those of phytohormones, GA, and auxin regulate plant cell elongation^43^,^44^. Significant differences in stem length as well as morphologies by microscopic analysis were noted at the 3rd and 4th nodes, between EOD and Cont. We propose that cell development in the transverse as well as longitudinal axes into the cortex and pith during early node formation was slowed by EOD treatment (Table 4). Thus, we suggest that stem elongation during the early developmental stages of *Eustoma* by EOD-FR light treatment involved elongation of the cell length along the longitudinal axis, especially into the cortex and pith during early node formation. A previous study showed that GA and auxin promote preferential cell growth along the longitudinal axis. GA mainly affects the elongation of young cells, and basic helix-loop-helix (bHLH) proteins of the *PRE* subfamily, such as PACLOBUTRAZOL RESISTANCE1 (PRE1), positively regulate organ elongation in *Arabidopsis* in response to GA^45^,^46^. Cell elongation regulated by the expression of *PRE* has also been demonstrated using a triantagonistic *bHLH* system, which involved activators for cell elongation (*ACE*) proteins that are activated by *PRE1* interacts with *IBH1* (ILI1 binding bHLH1) negatively regulated cell elongation^47^.In this study, DEGs annotated as transcription factor *bHLH* were also identified among DEGs related to circadian rhythm into KEGG pathway enrichment analysis (Supplementary Table S4), the expression levels of *bHLH 135* (CL7761) were higher in the stem of EOD than of Cont after EOD treatment (Fig. 4). Additionally, this DEG was highly homologous to encoding *PRE* gene. To our knowledge, research of about changes in expression pattern of transcription factor *bHLH* in *Eustoma* plants is lacking, and this is the first study to identify genes homologous to *PRE* that are trigged by EOD-FR treatment in other plants.

From the results in this study, we propose that increased expression of *GA20ox* and *YUCCA* involved in GA and auxin synthesis or that of *PIN* involved in auxin export, which are induced by EOD-FR light treatment, affects the elongation of cell length along the longitudinal axis at the nodes. Additionally, increased expression levels of DEGs annotated as transcription factor *bHLH* after EOD-FR light treatment is involved in cell elongation along the longitudinal axis at the nodes, subsequently resulting in stem elongation in the early developmental stages of *Eustoma*.

## Materials and Methods

### Seedling germination, planting, and EOD-FR light treatment

*E*. *grandiflorum* ‘Bolero White’ seeds (Miyoshi Co., Yamanashi, Japan) were sown on August 20, 2014, in plastic germination trays with 288 cells (21 mL/cell) filled with Metromix 350 (Sun Gro Horticulture Distribution Inc., USA). The seeded trays were maintained in a dark cool-room at 10 °C for 25 days. Then, the trays were transferred to a greenhouse and grown under constant temperature of 25 °C during the day (08:00–17:00) and 15 °C at night (17:00–08:00) for 6 weeks. Afterward, the seedlings were transferred to a plant box (60 cm long × 18 cm wide × 15 cm deep) on December 12, and grown at temperatures of over 18 °C. The seedlings were subjected to EDO-FR light treatment (EOD) from December 12, during 16:45–19:45 by using an FR light fluorescent lamp (Fuji Electric Co., Tottori, Japan). The sunrise and sunset times in the study region on December 12 were 07:02 and 16:51. Plants grown under ambient light without EOD-FR treatment were used as the Cont.

The number of nodes, node length, stem length, and fresh weights of above and belowground biomass per 10 replications of each treatment were measured on December 25. Stem and leaf of plants under both treatments were collected at 16:45, 19:45, and 22:45 on the same day, frozen immediately in liquid nitrogen, and stored at −80 °C until RNA extraction for RNA-seq and gene expression analysis.

### RNA extraction, library preparation, and RNA-seq

Total RNA was extracted from three biological replications of leaves of Cont and EOD collected at 19:45, which is end time of EOD treatment, on December 25 according to the methods described in Gasic *et al.*^48^, with some modifications, and an independent RNA pool was created for transcriptome analyses. Genomic DNA was eliminated from the total RNA preparation by using DNase I (New England Biolabs Inc.). The quality of total RNA was evaluated using an Agilent 2100 Bioanalyzer and Agilent RNA 6000 nano Kit (Agilent Technologies). Only samples with RNA integrity number (RIN) > 7.5 were used for RNA-seq. Library preparation was performed according to TruSeq RNA Sample Preparation v2 guide (Illumina). Oligo(dT) beads were used to isolate poly(A) mRNA from total RNA. Fragmentation buffer was used to obtain short mRNA fragments for use as templates to synthesize first-strand cDNAs by using random hexamer primers. Second-strand cDNAs were synthesized using DNA polymerase I (TaKaRa). Short fragments were purified using QiaQuick PCR extraction kit (Qiagen) and dissolved using EB buffer supplied in the kit, for end preparation and poly(A) addition. Afterward, the short fragments were connected using sequencing adaptors. Agarose gel electrophoresis was performed to select suitable fragments as templates for PCR amplification, and a library was prepared using Illumina HiSeq^TM^ 2000 by BGI. The read data were submitted to the DDBJ Read Archive (BioProject: PRJDB4037, BioSample: SSUB004508).

### Bioinformatics analysis

Raw reads obtained using the HiSeq 2000 were filtered to exclude low complexity reads and those containing adaptor sequences. The resulting clean reads were assembled using Trinity^49^, and TGICL^50^ were used to optimize the Trinity original assembly result by removing sequences that could not be extended on either end. Such sequences were defined as unigenes. Further, we aligned the unigenes to the NCBI non-redundant protein database by using BLASTx^51^ (E-value < 1e-7). The assembled unigenes were also annotated using Blast2GO^52^ with GO^53^ and KEGG^54^. After obtaining a GO annotation for every unigene, the WEGO online tool^55^ was used to classify GO functions for all the unigenes and to understand the distribution of gene functions of the species at the macro level. The calculation of unigene expression was based on the reads per kb per million reads (RPKM) method^56^. DEGs of EOD and Cont were selected according to the false discovery rate (FDR ≤ 0.001 and log_2_Ratio ≥ 1). DEG analysis was performed using the method described by Audic and Claverie^57^. The FDR^58^ was used to determine the P-value thresholds in multiple testing. DEGs were then subjected to GO functional enrichment analysis and KEGG pathway analysis. GO terms or KEGG pathways fulfilling Bonferroni correction-corrected P-value ≤ 0.05 were defined as significantly enriched GO terms/KEGG pathways in DEGs.

### Real-time PCR

Total RNA was used to synthesize first-strand cDNAs by using reverse transcriptase (TaKaRa). The cDNA was diluted 1/100 and used as a template for real-time PCR performed using a LightCycler 480 SYBR Green system (Roche Diagnostics, Basel, Switzerland). A total of 5 μL of diluted cDNA was added to 15 μL of the reaction mixture containing 3 μL of LightCycler 480 SYBR Green Mastermix and 0.5 mM of specific primer pairs for each gene (Supplementary Table S7) designed using the Primer Express software. The specificity of the amplification reaction for a given primer set was verified on the basis of their melting curves. Real-time PCR was performed as follows: an initial step of 2 min at 95 °C; followed by 45 cycles of 10 s at 95 °C, 20 s at 60 °C, and 20 s at 72 °C; melting for 0 s at 95 °C; and slow heating from 60 °C to 95 °C at 0.2 °C/s increments to confirm the amplification of single products. Relative expression was determined using the 2^-Δ Δ Ct^ algorithm by normalizing to the actin gene^59^, which did not show any differential expression in this study.

### Light microscopy

The collected stem samples were fixed in FAA, and dehydrated in an ethanol series (30, 50, 60, 70, 80, 90, 95, and 100%). The explants were then pre-infiltrated overnight with a mixture of equal parts of 100% ethanol and Technovit 7100 (Kulzer) base liquid and transferred to an infiltration solution of 100 ml Technovit 7100 base liquid and 1 g hardener I for 1 day. Afterward, these were embedded in Teflon molds with a mixture of 15 parts infiltration solution and one part hardener II. The samples were then polymerized for 1 h at room temperature and further for 6 h at 37 °C. Specimens were mounted on block-holders with Technovit 3040 (Kulzer), and tissue sections (1.5-μm thick) were cut using a sledge microtome (NS-31; Yamato). Microscopic analysis was performed using a Nikon Diaphot inverted microscope (Nikon) by observing cross and vertical sections of the pith and cortex of the stem.

## Additional Information

**How to cite this article**: Takemura, Y. *et al.* Gene expression changes triggered by end-of-day far-red light treatment on early developmental stages of Eustoma grandiflorum (Raf.) Shinn. *Sci. Rep.* **5**, 17864; doi: 10.1038/srep17864 (2015).

## Supplementary Material

Supplementary Information

Supplementary Table S1

## Figures and Tables

**Table 1:** Effect of EOD-FR treatment on growth in early developmental stages of *Eustoma grandiflorum*.

Treatment	Stem length (cm)	Number of nodes on main stem	Mean internode length (mm)	fresh weight on aerial part (g)	fresh weight on underground part (g)
Control	8.1	4.9	16.6	2.1	0.77
EOD-FR	13.4	5.1	27.1	2.4	0.75
T-test	** Z	NS	**	NS	NS

^Z^NS, *, or ** indicate non-significant, significant at p < 0.05, or 0.01, respectively. (*n* = 10).

**Table 2:** Summary of the sequencing and assembly.

Libraries^Z^	Control (1)	Control (2)	Control (3)	EOD-FR (1)	EOD-FR (2)	EOD-FR (3)	All
Total reads	56,397,212	66,135,068	77,080,190	62,863,124	62,960,474	71,124,618	
Total nucleotides	5,639,721,200	6,613,506,800	7,708,019,000	6,286,312,400	6,296,047,400	7,112,461,800	
GC percentage	44.16	44.16	44.29	44.07	43.92	44.06	
Contig	218,897	244,846	245,125	235,202	230,893	241,331	
Mean Length of contig (nt)	303	302	304	304	305	304	
Contig N50	483	471	479	483	486	478	
Unigene	91,162	105,911	105,737	100,700	100,155	103,437	161,051
Mean Length of unigene (nt)	1,013	1,042	1,025	1,042	1,033	1,034	1,340
Unigene N50	1,784	1,849	1,827	1,841	1,825	1,831	2,242
Distinct Clusters	44,242	50,677	50,756	48,511	48,605	49,835	84,563
Distinct Singletons	46,920	55,234	54,981	52,189	51,550	53,602	76,488

^Z^6 libraries as biological replicates constructed from the floral buds of each sample number. Number of replicates shown in brackets.

**Table 3:** Top 5 of GO terms in DEG set up-regulated by EOD-FR treatment.

GO term	UP	Down
**Biological process**		
oxidation-reduction process	36	34
transmembrane transport	27	15
regulation of transcription, DNA-dependent	19	1
proximal/distal pattern formation	14	0
flower morphogenesis	14	0
floral organ abscission	14	0
response to auxin	14	2
**Molecular function**		
sequence-specific DNA binding transcription factor activity	24	5
ATP binding	23	22
oxidoreductase activity	20	10
calmodulin binding	15	0
transporter activity	14	6
**Cellular Component**		
nucleus	66	34
plasma membrane	61	66
integral component of membrane	43	21
cytoplasm	42	8
membrane	27	19

**Table 4:** Morphology of internode of the main stem in early developmental stages of *Eustoma grandiflorum* (Vertical section).

Node	Treatment	Pith diameter (mm)	Cortex diameter (mm)	Width of cell (μm)		Length of cell (μm)	
				Pith	Cortex	Pith	Cortex
	Control	1.54	0.23	8.65	1.59	16.50	6.89
4th-node	EOD-FR	1.22	0.18	7.18	1.36	24.34	7.91
	T-test	NS^Z^	NS	**	**	**	NS
	Control	1.43	0.21	10.49	0.99	24.26	4.00
3rd-node	EOD-FR	1.50	0.21	7.99	1.27	25.82	8.07
	T-test	NS	NS	**	**	NS	**

^Z^NS, *, or ** indicate non-significant, significant at p < 0.05, or 0.01, respectively. (*n* = 5).

15 cells were measured in each stems for calculating width and length of cell.
