# Supplementary material for: Gene expression changes triggered by end-of-day far-red light treatment on early developmental stages of Eustoma grandiflorum (Raf.) Shinn
Source: Sci Rep. 2015 Dec 8;5:17864. doi: 10.1038/srep17864 (PMC4672308; doi:10.1038/srep17864)
Supplement: Supplementary Information [file srep17864-s1.pdf]

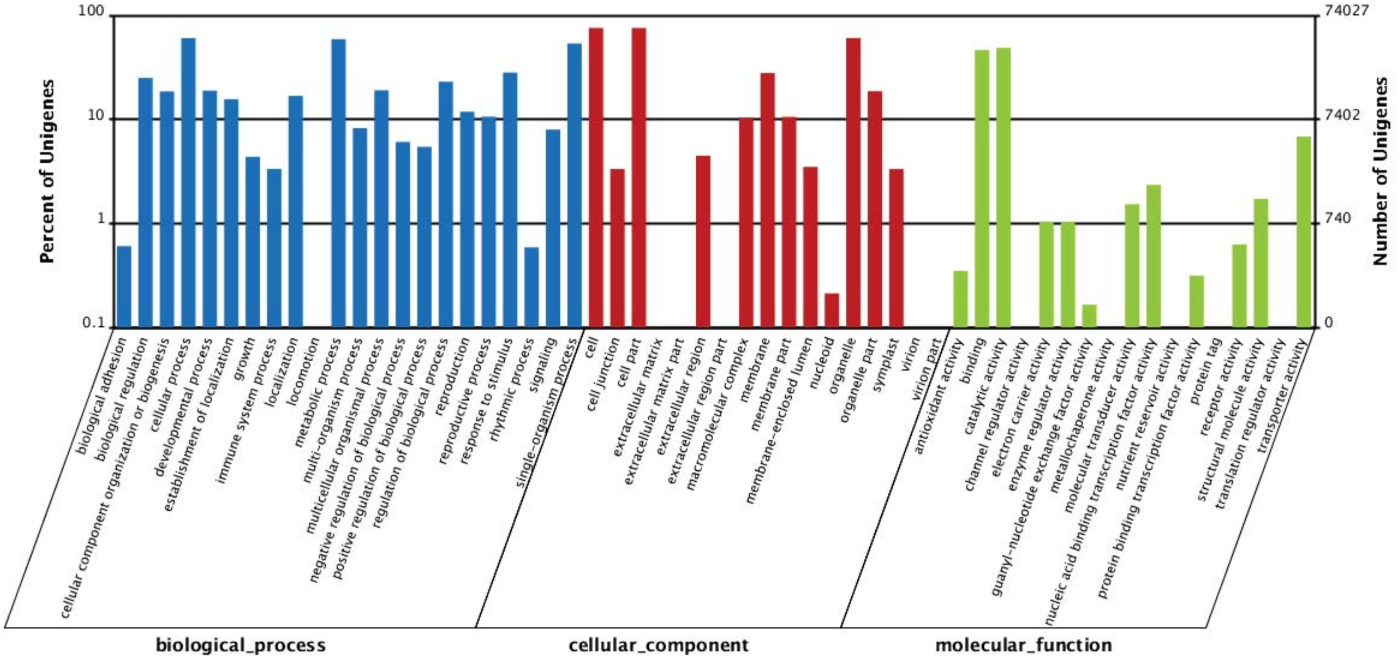

Supplementary Fig. S1. GO assignment of all unigenes. The unigenes were mapped to three main categories: biological process, cellular component and molecular function. The right hand y-axis indicates the number of annotated unigenes.

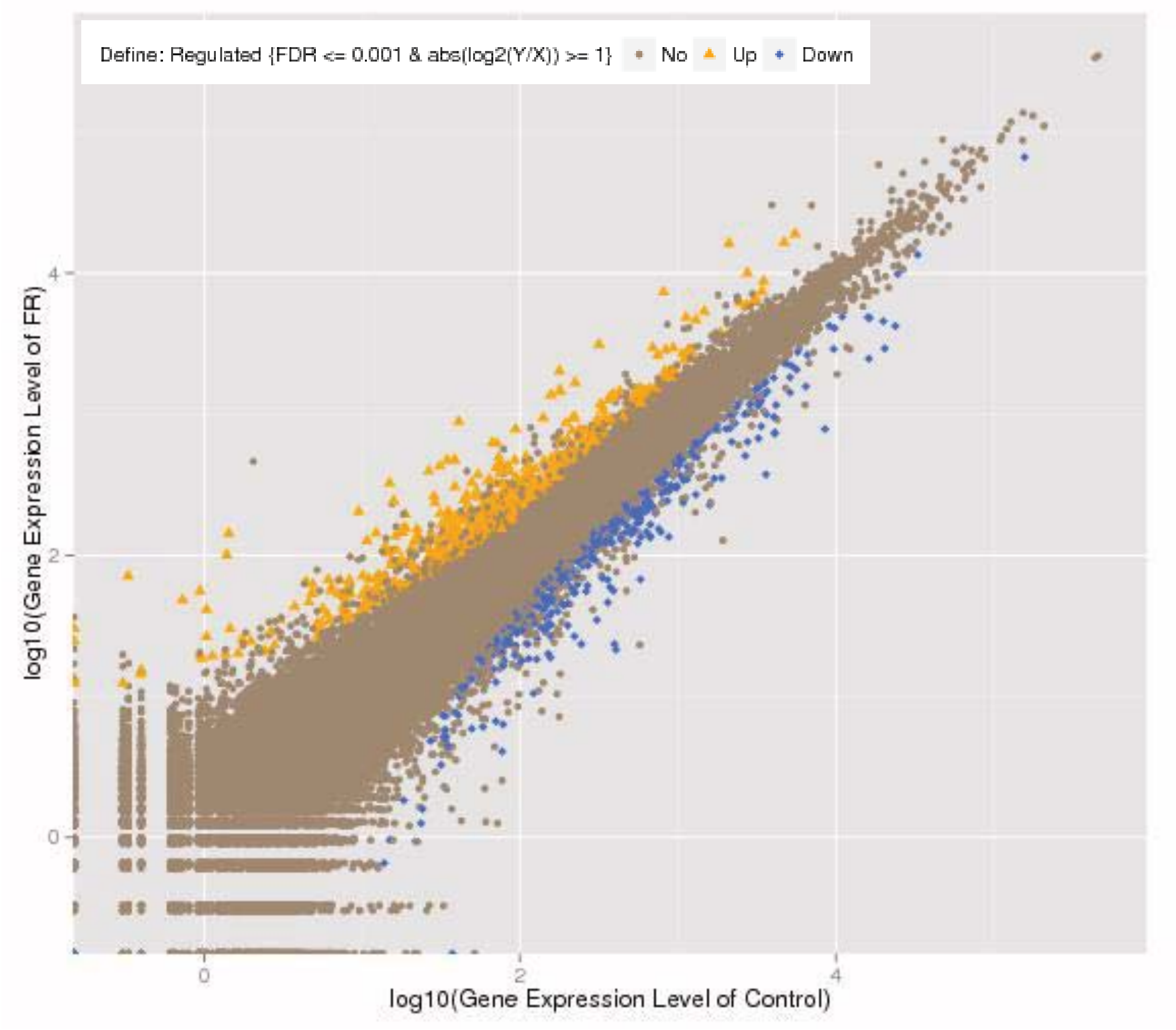

Supplementary Fig. S2. Differentially expressed genes in the EOD-FR sample (EOD) compared with those in the control sample (Cont). The significantly up-regulated genes by EOD-FR treatment were marked in yellow while the significantly down-regulated genes were marked in blue with the threshold of FDR ≦ 0.001 and (log_2Ratio) ≧ 1.

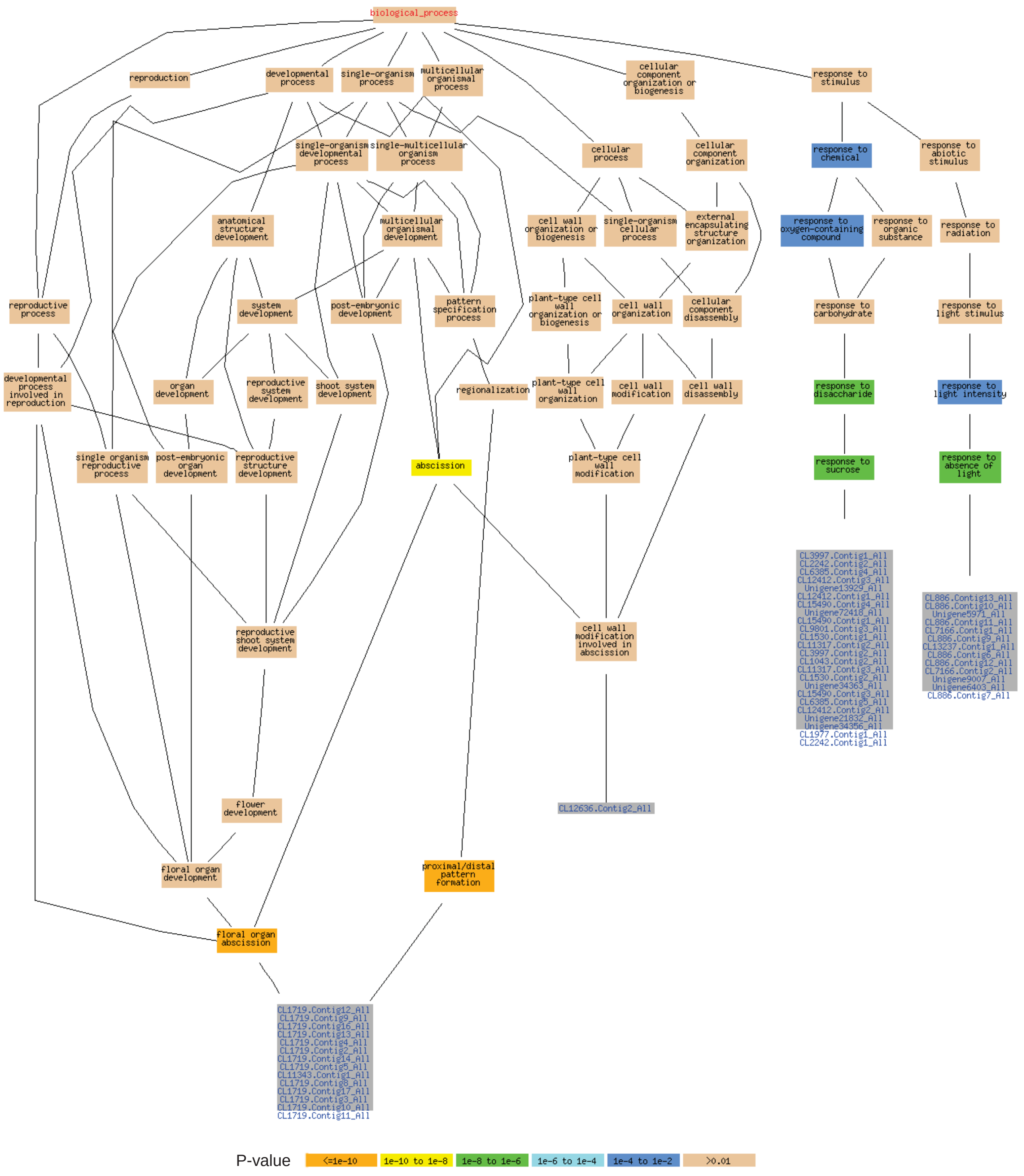

Supplementary Fig. S3A. Enriched GO terms in category of 'biologycal process' of differential expressed genes between EOD and Cont plants in GO enrichment analysis.

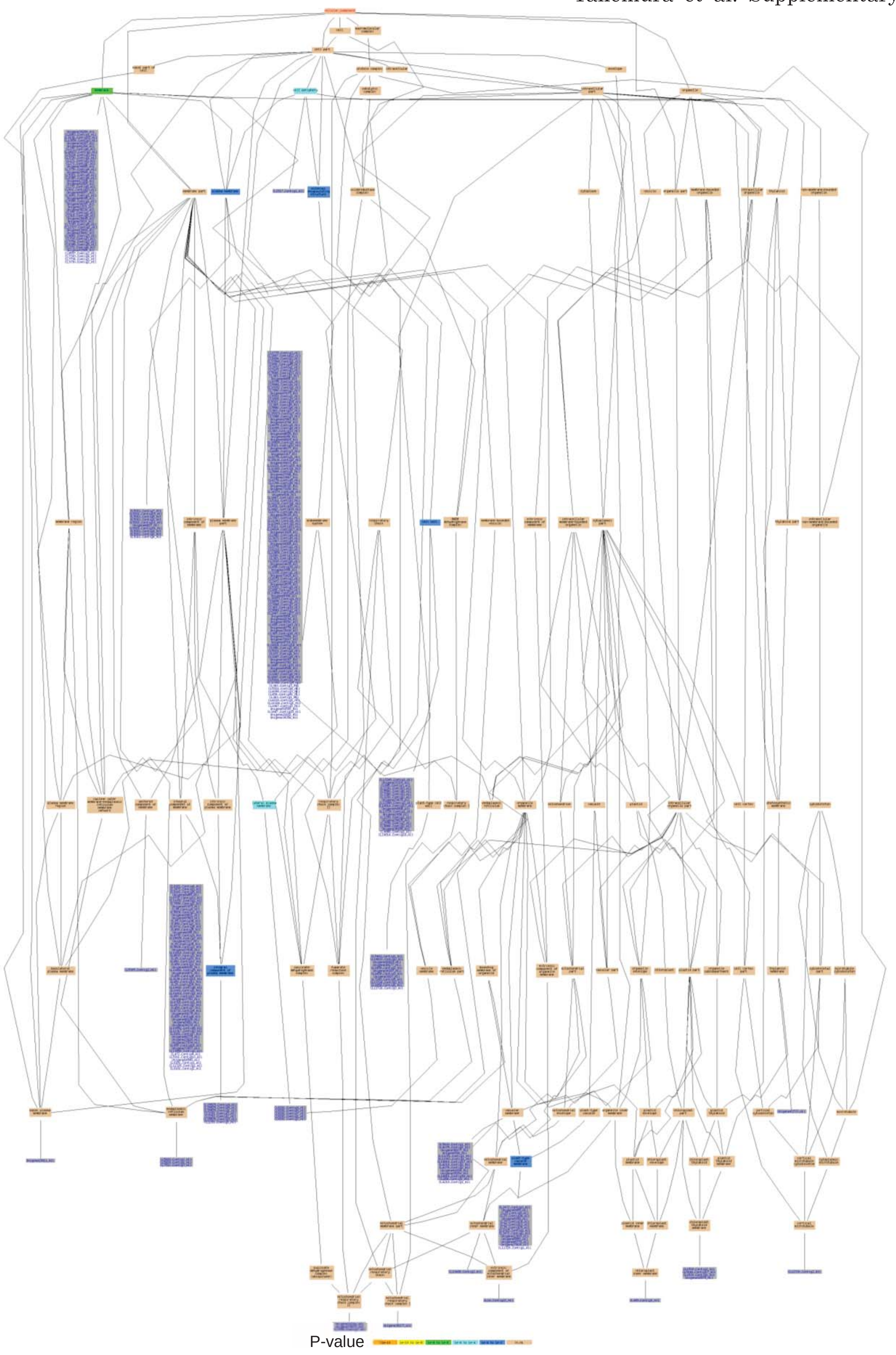

Supplementary Fig. S3B. Enriched GO terms in category of 'cellular component' of differential expressed genes between EOD and Cont plants in GO enrichment analysis.

Takemura et al. Supplementary Fig.S3

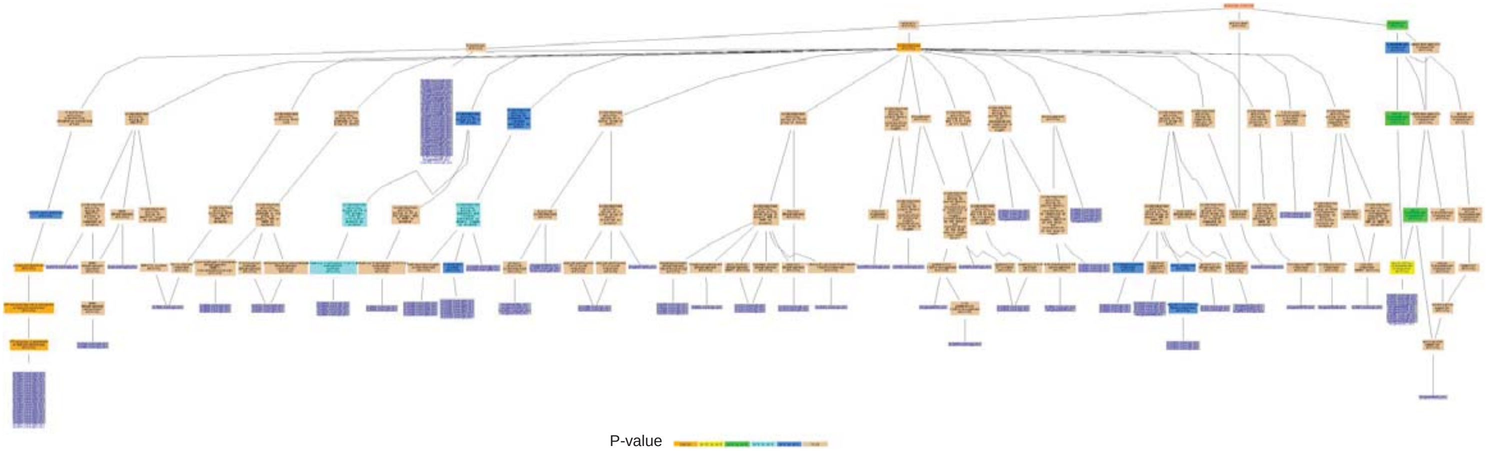

Supplementary Fig. S3C. Enriched GO terms in category of 'molecular function' of differential expressed genes between EOD and Cont plants in GO enrichment analysis.

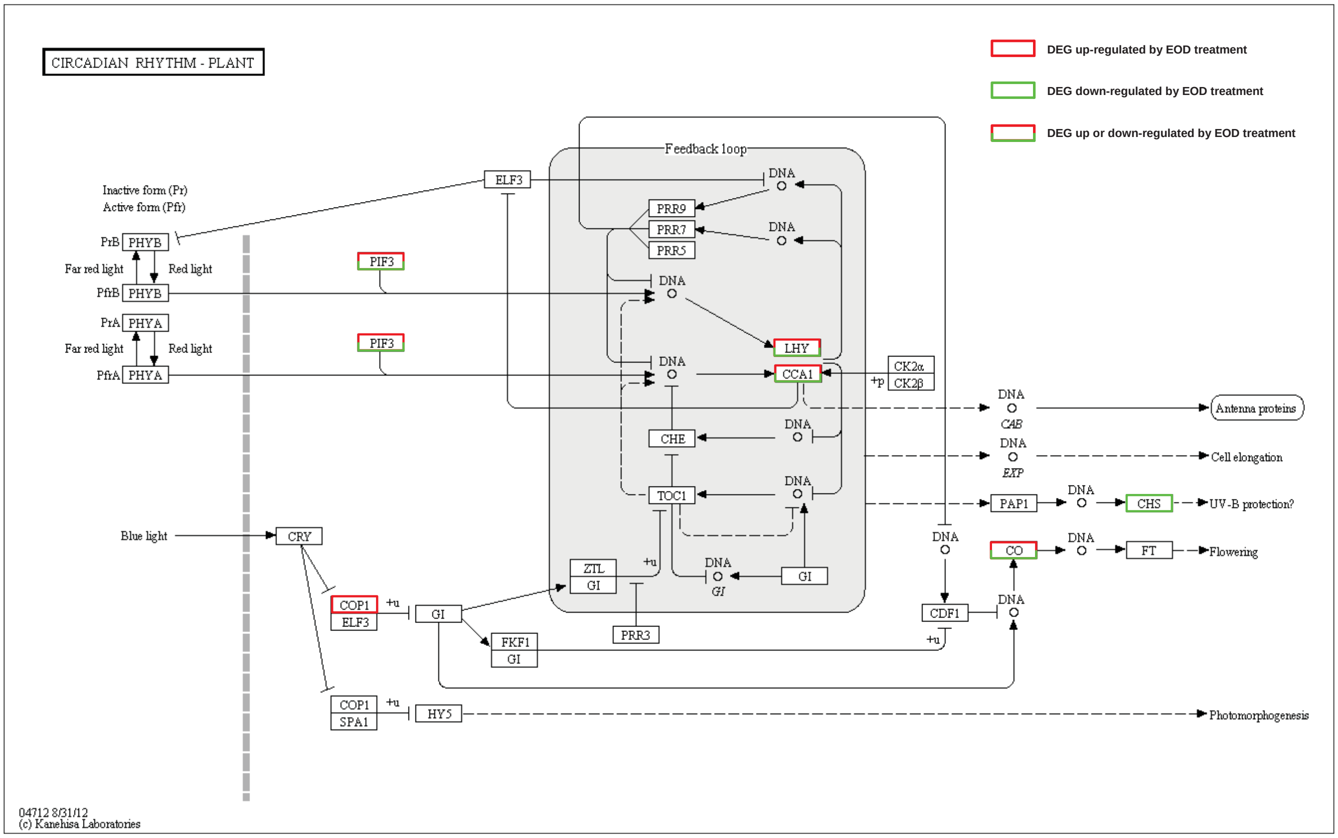

Supplementary Fig. S4A. Circadian rhythm - plant' KEGG pathway enriched in this study.

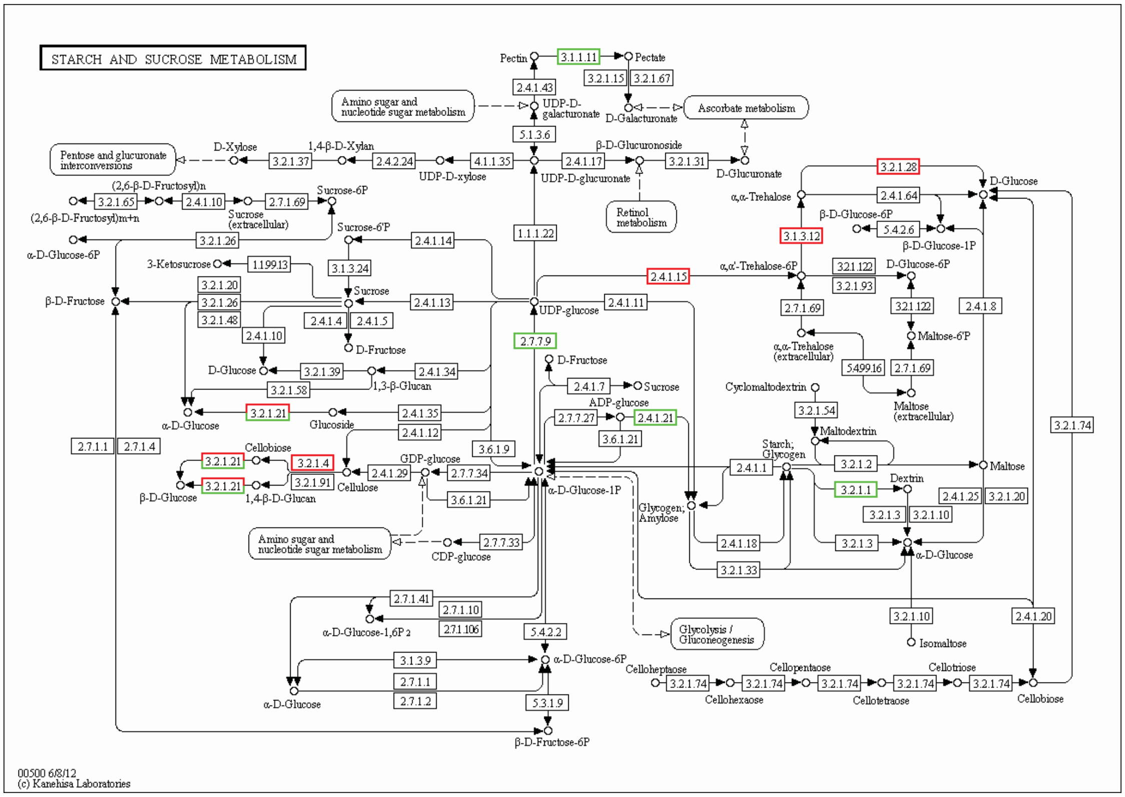

DEG up-regulated by EOD treatment

DEG down-regulated by EOD treatment

DEG up or down-regulated by EOD treatment

Supplementary Fig. S4B. Starch and sucrose metabolism' KEGG pathway enriched in this study.

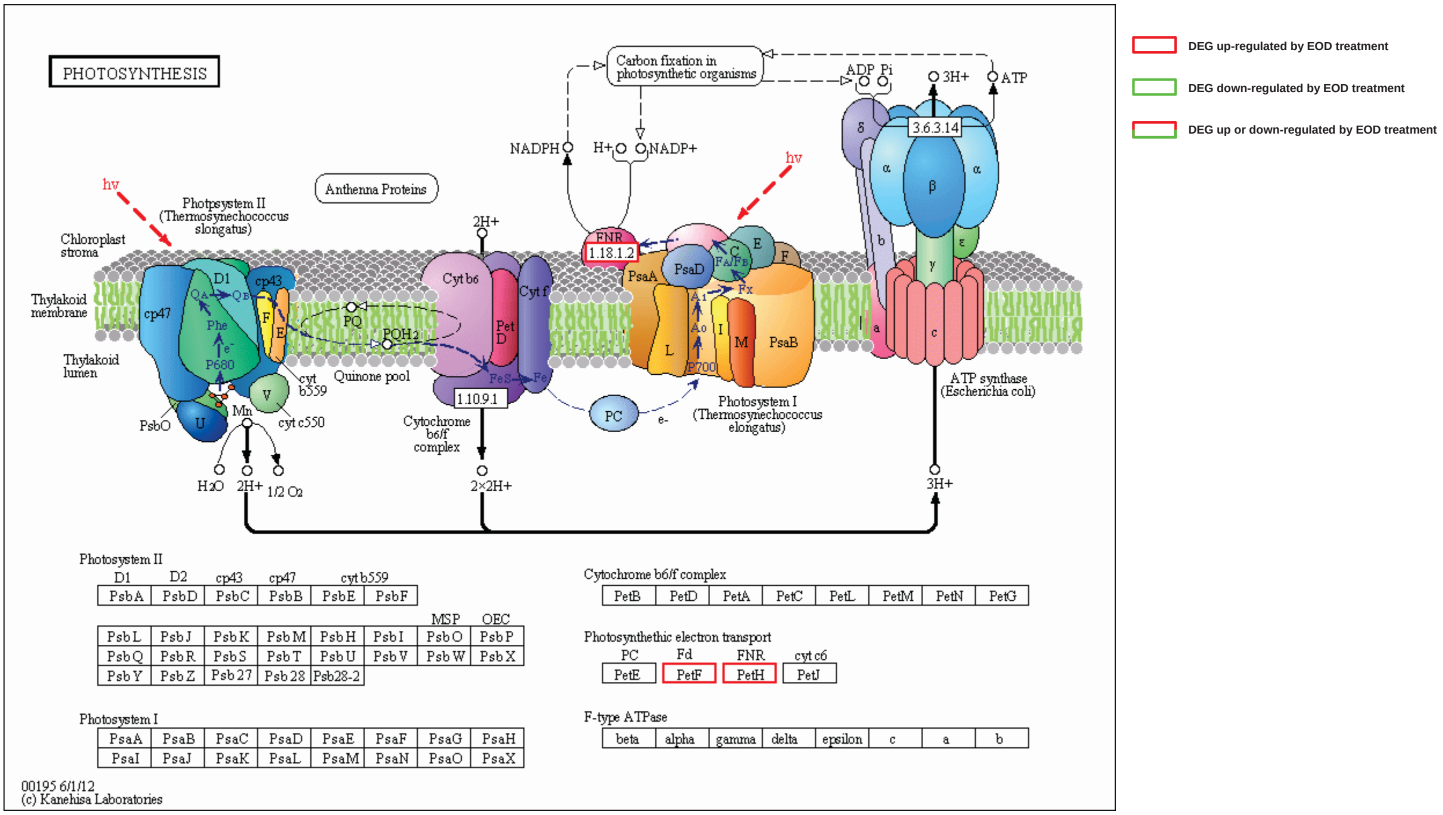

Supplementary Fig. S4C. Photosynthesis' KEGG pathway enriched in this study.

DEG up-regulated by EOD treatment

DEG down-regulated by EOD treatment

DEG up or down-regulated by EOD treatment

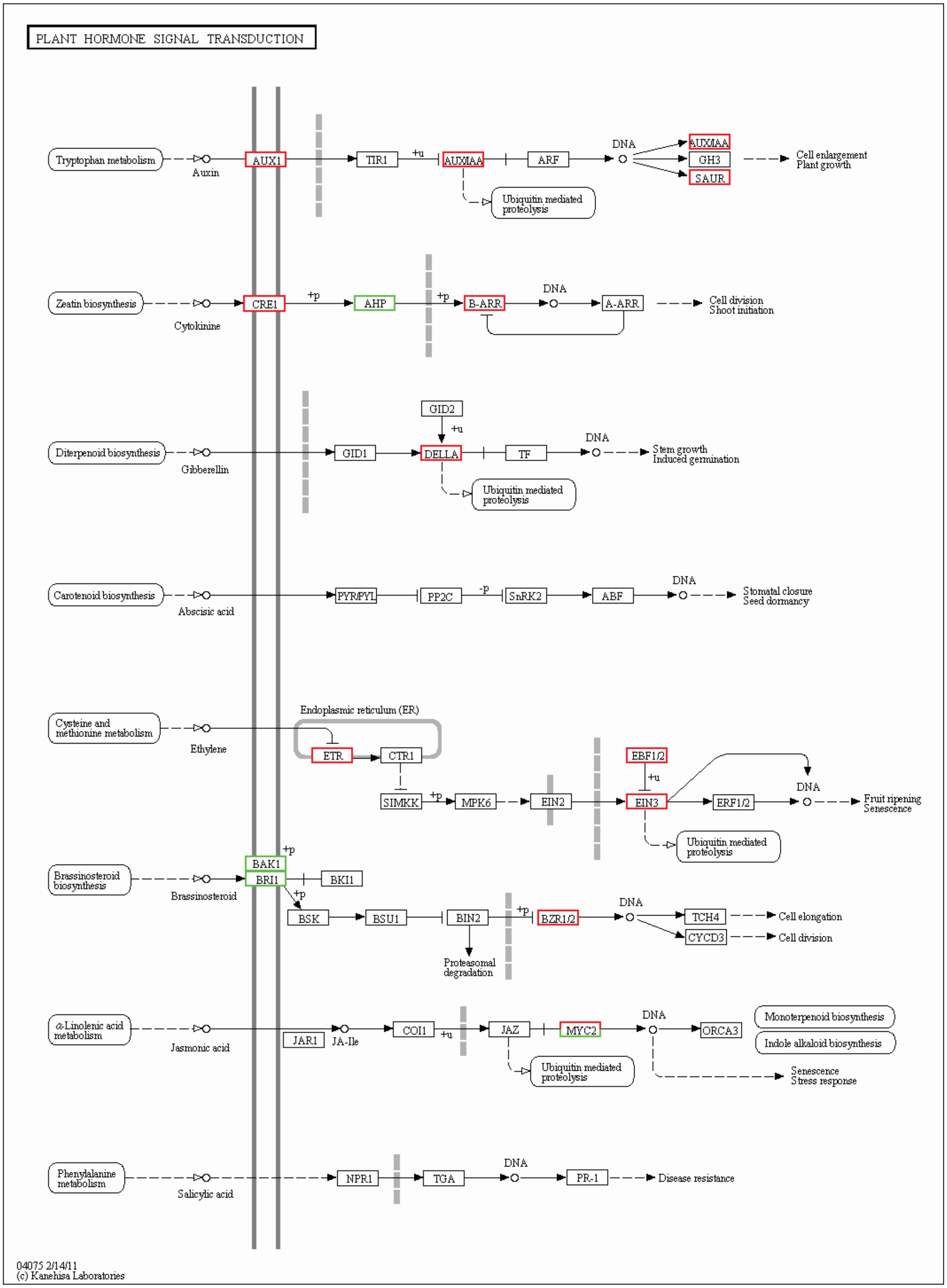

Supplementary Fig. S4D. Plant hormone signal transduction' KEGG pathway enriched in this study.

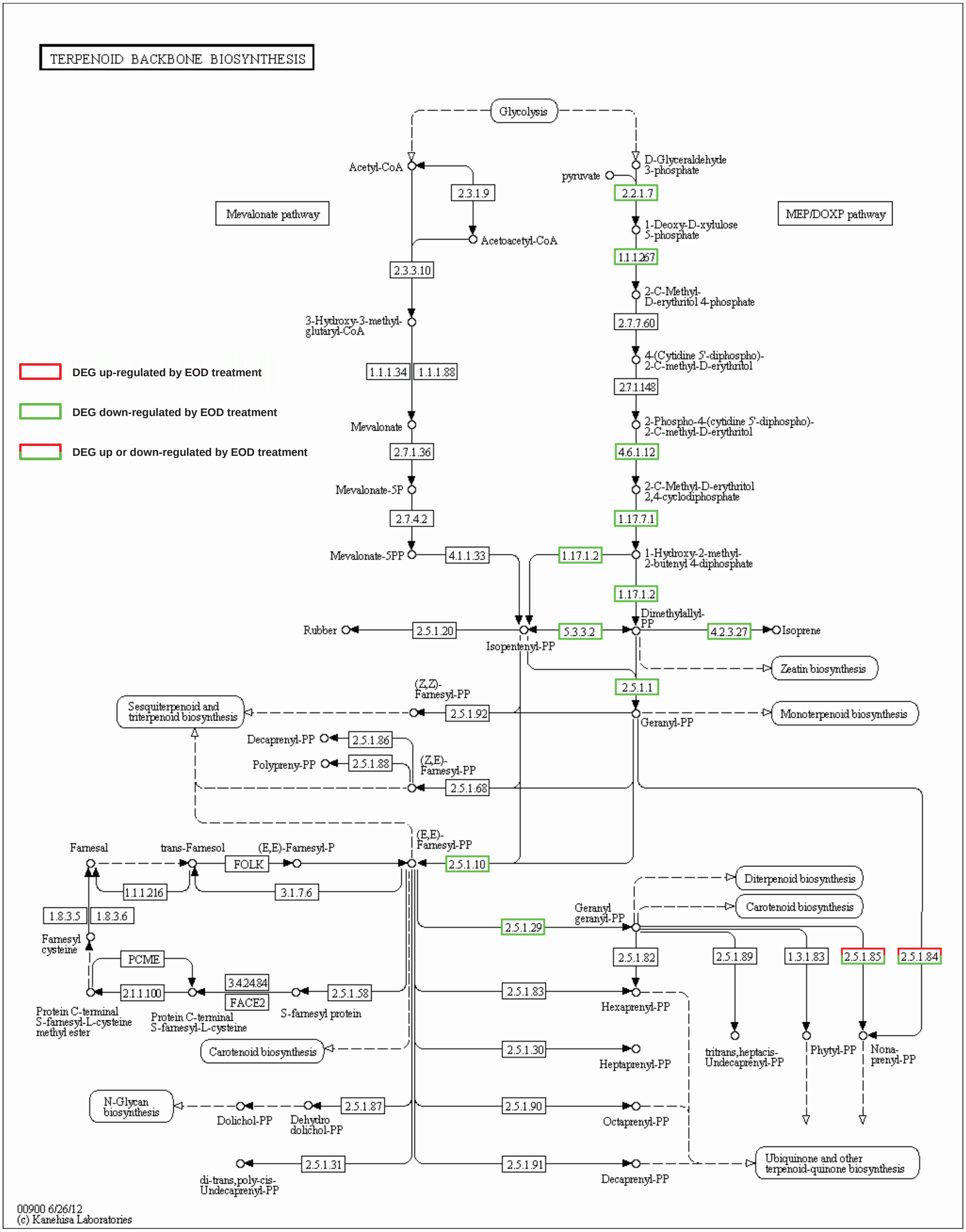

Supplementary Fig. S4E. Terpenoid backbone biosynthesis' KEGG pathway enriched in this study.

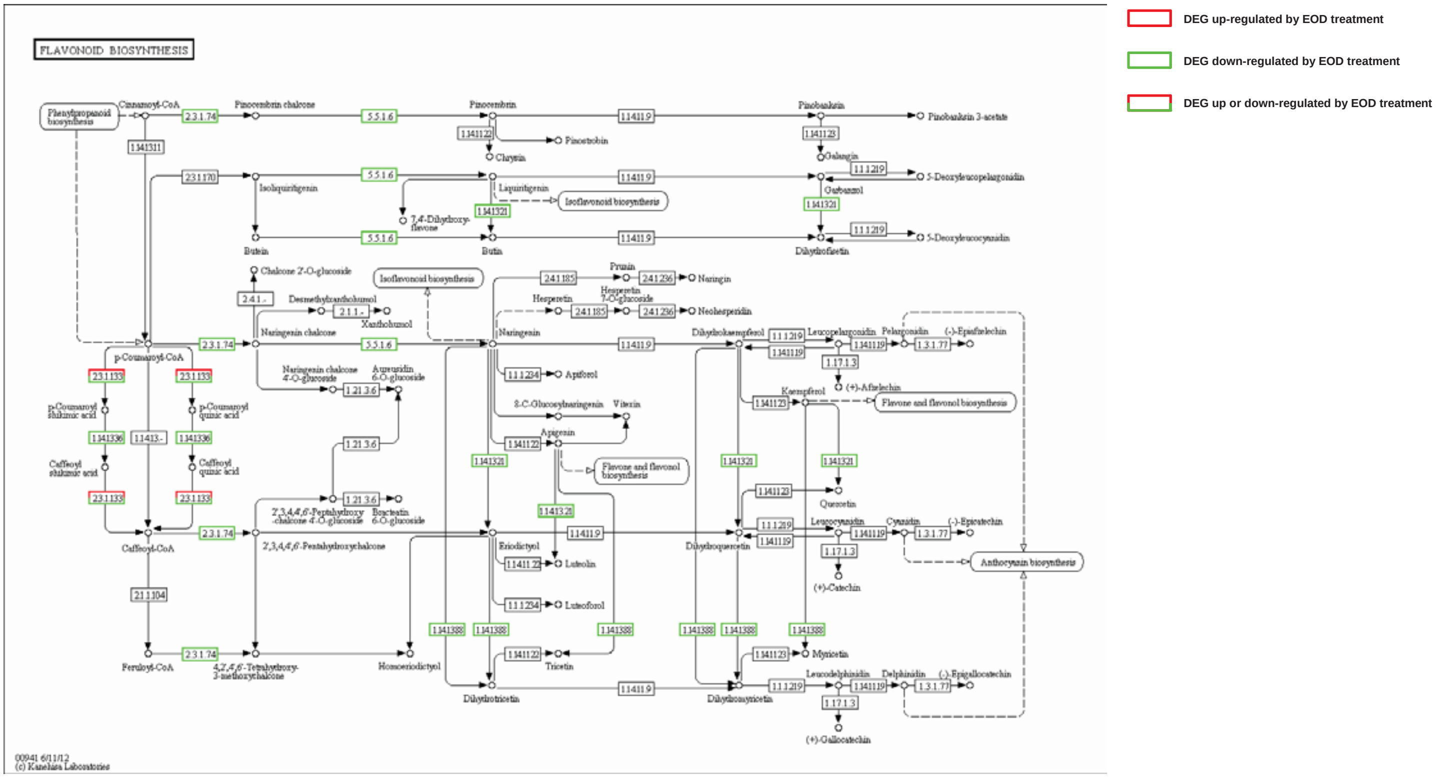

Supplementary Fig. S4F. Flavonoid biosynthesis' KEGG pathway enriched in this study.

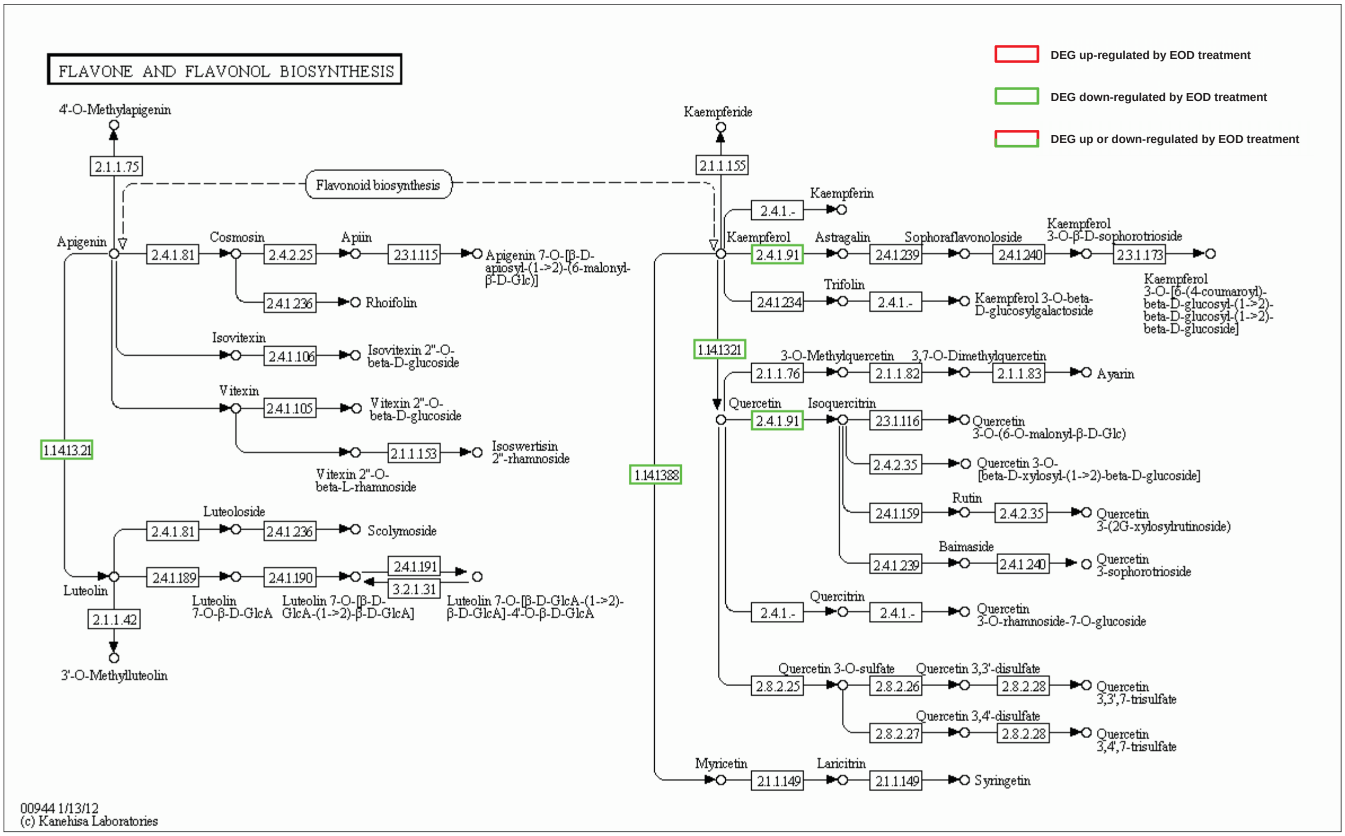

Supplementary Fig. S4G. Flavone and flavonol biosynthesis' KEGG pathway enriched in this study.

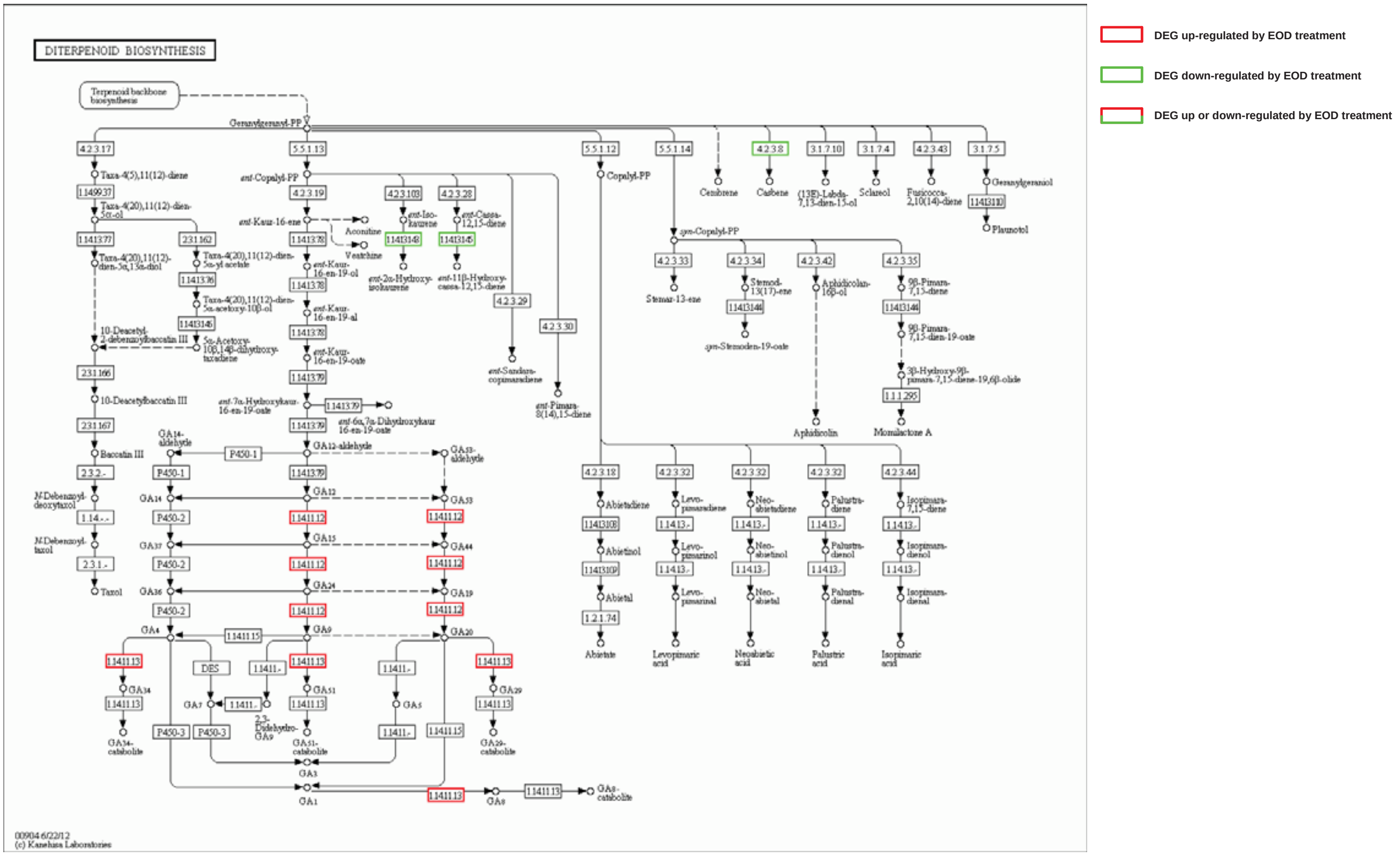

Supplementary Fig. S4H. Diterpenoid biosynthesis' KEGG pathway enriched in this study.

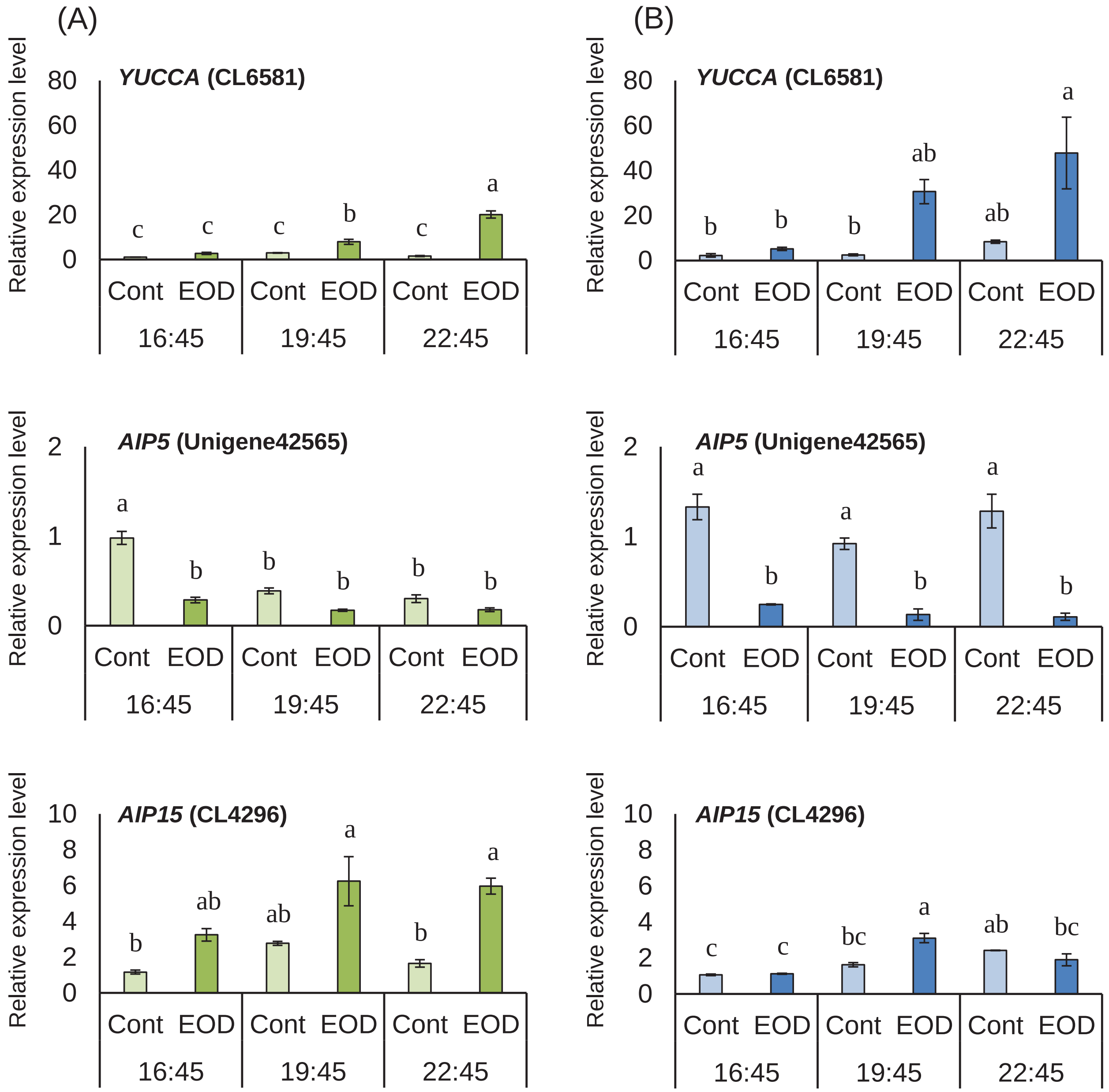

Supplementary Fig. S5. Relative expression levels of DEGs annotated as genes related to synthesis or signaling of auxin in leaf (A) and stem (3rd- and 4th-nodes) (B) of *Eustoma grandiflorum* collected on December 25. Different letters within the same column show a significant difference by Tukey-Kramer's HSD tests at the 5% level.

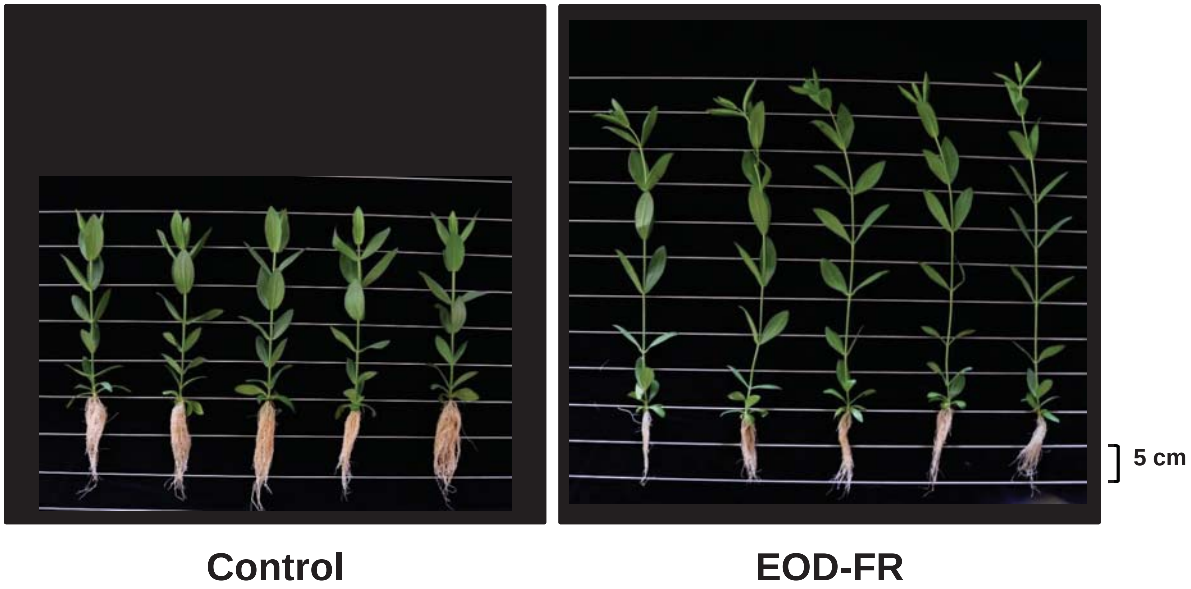

Supplementary Fig. S6. Photographs in mid developmental stages of *Eustoma grandiflorum* treated in this study. *Eustoma* plants in photographs were collected on January 22 as mid developmental stages.

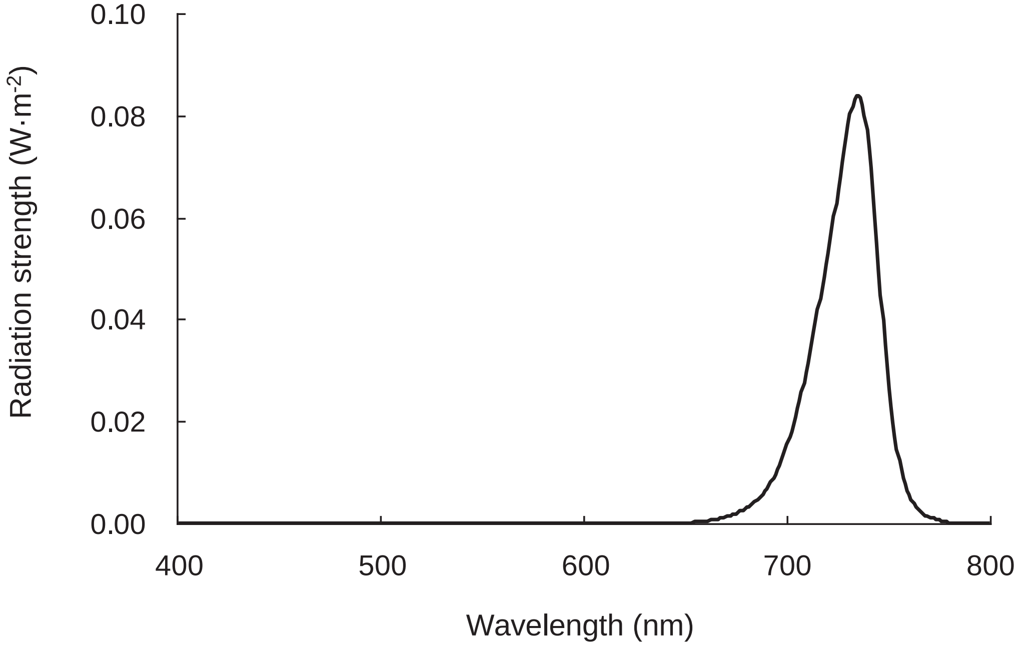

Supplementary Fig. S7. Spectrum distribution characteristics of light sources used to study.

Supplementary Table S2A. List of GO enrichment analysis results: category 'biologycal process' (P-value < 1E-05).

Gene Ontology term	Cluster frequency (No. of DEGs (% of 547))	Corrected P-value
proximal/distal pattern formation	14 (2.6%)	9.44E-15
floral organ abscission	14 (2.6%)	4.31E-11
abscission	15 (2.7%)	4.69E-10
response to absence of light	13 (2.4%)	1.96E-08
response to sucrose	24 (4.4%)	8.14E-08
response to disaccharide	24 (4.4%)	8.14E-08
chloride transport	12 (2.2%)	2.12E-07
auxin efflux	11 (2.0%)	1.36E-06
inorganic anion transport	23 (4.2%)	7.30E-06
embryonic root morphogenesis	5 (0.9%)	7.69E-05
root hair initiation	5 (0.9%)	7.69E-05

Supplementary Table S2B. List of GO enrichment analysis results: category 'cellular component' (P-value < 1E-05).

Gene Ontology term	Cluster frequency (No. of DEGs (% of 511))	Corrected P-value
membrane	242 (47.4%)	3.36E-07
cell periphery	160 (31.3%)	2.50E-06
lateral plasma membrane	5 (1.0%)	6.50E-06

Supplementary Table S2C. List of GO enrichment analysis results: category 'molecular function' (P-value < 1E-05).

Gene Ontology term	Cluster frequency (No. of DEGs (% of 486))	Corrected P-value
UTP:glucose-1-phosphate uridylyltransferase activity	20 (4.1%)	2.30E-28
UTP-monosaccharide-1-phosphate uridylyltransferase activity	20 (4.1%)	8.43E-28
uridylyltransferase activity	20 (4.1%)	8.91E-27
oxidoreductase activity	108 (22.2%)	1.30E-12
auxin efflux transmembrane transporter activity	11 (2.3%)	4.25E-10
auxin transmembrane transporter activity	12 (2.5%)	1.47E-08
voltage-gated chloride channel activity	12 (2.5%)	2.15E-08
chloride channel activity	12 (2.5%)	2.15E-08
chloride transmembrane transporter activity	12 (2.5%)	3.95E-08
efflux transmembrane transporter activity	11 (2.3%)	6.21E-08
voltage-gated anion channel activity	12 (2.5%)	8.74E-08
anion channel activity	12 (2.5%)	1.08E-07
transporter activity	87 (17.9%)	5.45E-07
voltage-gated ion channel activity	16 (3.3%)	6.00E-07
voltage-gated channel activity	16 (3.3%)	6.00E-07
acid phosphatase activity	13 (2.7%)	9.14E-07
4-hydroxy-3-methylbut-2-en-1-yl diphosphate synthase activity	5 (1.0%)	2.17E-06
oxidoreductase activity, acting on CH or CH2 groups, with an iron-sulfur protein as acceptor	5 (1.0%)	2.16E-05
nitrate transmembrane transporter activity	7 (1.4%)	4.62E-05
gated channel activity	16 (3.3%)	6.98E-05
oxidoreductase activity, acting on diphenols and related substances as donors, oxygen as acceptor	9 (1.9%)	9.66E-05
substrate-specific channel activity	17 (3.5%)	9.72E-05

Table S3. Highly enriched KEGG classes of DEGs (P-value < 1E-05).

Pathway	No. of DEGs with pathway annotation (% of 577)	P-value	Pathway ID
Circadian rhythm - plant	35 (6.07%)	2.38E-22	ko04712
Biosynthesis of secondary metabolites	125 (21.66%)	6.28E-17	ko01110
Metabolic pathways	184 (31.89%)	1.33E-13	ko01100
Pentose and glucuronate interconversions	25 (4.33%)	1.90E-13	ko00040
Starch and sucrose metabolism	39 (6.76%)	1.23E-11	ko00500
Galactose metabolism	22 (3.81%)	2.75E-11	ko00052
Sphingolipid metabolism	15 (2.60%)	1.42E-08	ko00600
Amino sugar and nucleotide sugar metabolism	23 (3.99%)	5.75E-08	ko00520
Stilbenoid, diarylheptanoid and gingerol biosynthesis	12 (2.08%)	6.22E-08	ko00945
Terpenoid backbone biosynthesis	32 (5.55%)	1.30 E-07	ko00900
Flavonoid biosynthesis	13 (2.25%)	7.01E-07	ko00941
Pantothenate and CoA biosynthesis	10 (1.73%)	3.59E-05	ko00770
Diterpenoid biosynthesis	10 (1.73%)	3.89E-05	ko00904
Phenylpropanoid biosynthesis	19 (3.29%)	4.67E-05	ko00940

Supplementary Table S4. Differentially expressed genes related to circadian rhythm in EOD-FR treatment of *Eustoma grandiflorum*.

Gene ID	Length (bp)	log2 (EOD-FR/Control)	P-value	Annotation
PIF3				
CL10572.Contig2	1,808	4.44	9.22E-51	predicted protein [Populus trichocarpa]
CL5482.Contig3	2,906	1.33	2.51E-06	PREDICTED: transcription factor bHLH130-like [Solanum lycopersicum]
CL2342.Contig2	2,017	1.14	1.61E-07	hypothetical protein PRUPE_ppa005829mg [Prunus persica]
CL176.Contig7	4,175	−1.08	1.57E-08	PREDICTED: transcription factor bHLH48-like [Solanum lycopersicum]
CL10572.Contig2	1,808	4.44	9.22E-51	predicted protein [Populus trichocarpa]
LHY or CCA1				
CL6114.Contig24	2,805	1.47	5.67E-10	MYB transcription factor, partial [Catharanthus roseus]
CL6114.Contig17	2,710	1.33	4.62E-06	putative At5g37260 [Solanum ochranthum]
Unigene50471	728	−2.97	2.45E-06	protein CCA1-like, partial [Cucumis sativus]
CL1022.Contig2	2,400	−2.29	1.68E-15	PREDICTED: protein LHY-like [Vitis vinifera]
Unigene14429	1,175	−1.78	2.80E-13	predicted protein [Populus trichocarpa]
Unigene11465	984	−1.56	3.10E-15	predicted protein [Populus trichocarpa]
COP1				
CL11693.Contig1	3,103	3.94	4.04E-06	PREDICTED: uncharacterized protein LOC101268835 [Solanum lycopersicum]
CO				
CL7349.Contig1	1,876	3.05	1.90E-12	PREDICTED: zinc finger protein CONSTANS-LIKE 5-like [Vitis vinifera]
Unigene24442	779	−2.14	7.99E-20	PREDICTED: probable salt tolerance-like protein At1g78600-like [Vitis vinifera]
CL6195.Contig2	661	−2.09	1.59E-07	PREDICTED: probable salt tolerance-like protein At1g78600-like [Vitis vinifera]
CHS				
Unigene15251	1,424	−1.52	7.81E-08	naringenin-chalcone synthase [Prunus avium]

These genes were picked up after KEGG pathway enrichment.

Supplementary Table S5. Morphology of internode of the main stem in early developmental stages of *Eustoma grandiflorum* (Cross section).

Node	Treatment	Pith		Cortex	
		Pith area (mm^2)	Number of cells	Cortex area (mm^2)	Number of cells
4th-node	Control	1.87	703	0.69	1831
	EOD-FR	1.21	586	0.40	1803
	T-test	NS [Z]	NS	*	NS
3rd-node	Control	1.61	480	0.59	1551
	EOD-FR	1.77	545	0.60	1620
	T-test	NS	NS	NS	NS

[Z] NS, *, or ** indicate non-significant, significant at $p < 0.05$, or 0.01, respectively. ($n = 5$).

Supplementary Table S6. Effect of EOD-FR treatment on growth in mid developmental stages of *Eustoma grandiflorum*.

Treatment	Stem length (cm)	Number of nodes on main stem	Mean internode length (mm)	fresh weight on aerial part (g)	fresh weight on underground part (g)
Control	21.4	8.0	26.8	5.3	1.54
EOD-FR	43.2	9.4	46.0	6.0	1.15
T-test	** [Z]	**	**	NS	NS

[Z] NS, *, or ** indicate non-significant, significant at $p < 0.05$, or 0.01, respectively. ($n = 10$).

Supplementary Table S7. Primers used for real-time PCR.

Target gene	Primer sequence (5' to 3')	Product size (bp)
BTB/POZ 1 (CL1719)	ATATTCAAAGCTGATGCTCAGTGG<br>GAGAGGAGAAGAAGGTAGAGGGAGA	141
BTB/POZ 2 (CL11343)	ACAAACACCAGCCCAGCA<br>GGACAAAGAGACAGAGTAAGGAGGA	147
ABCB transporter (Unigene72607)	GCCTGCCTCTCTCCAGTTCTT<br>ATGCGATTTTGCTCTGCTACAC	91
PIN 4 (CL6181)	CCGGCAGATGCATTAGGA<br>TTGGAGGTGGTGGGAGAGA	112
YUCCA (CL6581)	AGCGTGTGGTGCCTGAAA<br>TCCGCATCCCACAACAAG	119
AIP 5 (Unigene42565)	AACACTGGGCCTTTTCTCCTC<br>CGGCTGGAACCCTATTTTACTATCT	105
AIP 15 (CL4296)	TTCTCCCCTACATACACAGCAAAA<br>AACAAACTGCATCAAGCTCCAA	137
GA20ox (CL10815)	ATCACTTGCCTTCTTTCTTTGTCC<br>CATGTCGGCTCTGTAATGCTTC	148
GA2ox (CL14653)	CCATAAGGCAAGGCAAAGGA<br>AAATGGAAGGTTGAAGAGTGTGAAG	125
bHLH 157 (CL2504)	TTCACACAGGACCTCTATCAGCA<br>ATCCGTAAAGAAAATCCCAGCA	135
bHLH 135 (CL7761)	AACGTGATCTTCGGCTAGACATT<br>CCCCTTCTTGCTTTCACATCTT	135
bHLH 130 (CL5482)	CAACATCATTCCAGCCAAAATC<br>CGTCTTCCCTATCACTCACTCTACC	84
bHLH 63 (CL2342)	CAGCGGAAAAGCAAATGAAA<br>CGAGCACAAAACCGAACC	120
Actin	TCTCTATGCTAGTGGTCGAA<br>CTCTCGGTGAGGATCTTC	165
